# An Update on the General Features of Breast Cancer in Male Patients—A Literature Review

**DOI:** 10.3390/diagnostics12071554

**Published:** 2022-06-26

**Authors:** Sinziana Ionescu, Alin Codrut Nicolescu, Marian Marincas, Octavia-Luciana Madge, Laurentiu Simion

**Affiliations:** 1 1st Clinic of General Surgery and Surgical Oncology, Bucharest Oncology Institute, 022328 Bucharest, Romania; sinzianaionescu30@gmail.com (S.I.); dr.simion.laurentiu@gmail.com (L.S.); 2 Department of Surgery, “Carol Davila” University of Medicine and Pharmacy, 050474 Bucharest, Romania; 3 Roma Medical Center for Diagnosis and Treatment, 011774 Bucharest, Romania; 4 Faculty of Letters, University of Bucharest, 050663 Bucharest, Romania

**Keywords:** male, breast, cancer, diagnosis, treatment, prognosis

## Abstract

Male breast cancers are uncommon, as men account for less than 1 percent of all breast carcinomas. Among the predisposing risk factors for male breast cancer, the following appear to be significant: (a) breast/chest radiation exposure, (b) estrogen use, diseases associated with hyper-estrogenism, such as cirrhosis or Klinefelter syndrome, and (c) family health history. Furthermore, there are clear familial tendencies, with a higher incidence among men who have a large number of female relatives with breast cancer and (d) major inheritance susceptibility. Moreover, in families with BRCA mutations, there is an increased risk of male breast cancer, although the risk appears to be greater with inherited BRCA2 mutations than with inherited BRCA1 mutations. Due to diagnostic delays, male breast cancer is more likely to present at an advanced stage. A core biopsy or a fine needle aspiration must be performed to confirm suspicious findings. Infiltrating ductal cancer is the most prevalent form of male breast cancer, while invasive lobular carcinoma is extremely uncommon. Male breast cancer is almost always positive for hormone receptors. A worse prognosis is associated with a more advanced stage at diagnosis for men with breast cancer. Randomized controlled trials which recruit both female and male patients should be developed in order to gain more consistent data on the optimal clinical approach.

## 1. Introduction

Female breast cancer is the most frequently diagnosed tumor and one of the leading causes of cancer-related mortality worldwide. Male breast cancer is uncommon, representing less than one percent of all breast cancers. It is more prevalent in elderly men and resembles postmenopausal breast cancer in its behavior, according to various studies, including that of Garreffa [1]. However, the incidence is increasing, reaching 15 percent in some patient groups over the course of their lives. A study by Abdekwahab Yousef [2] reports that age, hormonal imbalance, radiation exposure, and a family history of breast cancer are the most significant risk factors for the development of male breast cancer. Instances of the latter can be linked to mutations in genes with high or low penetrance. A BRCA2 gene mutation is the most important risk factor for the development of male breast cancer.

The majority of cases are diagnosed late due to a lack of awareness of the existence of this cancer in males and ignorance of the associated risk factors.

In addition to the above-mentioned issues, men with breast cancer have an elevated risk of developing a second cancer.

## 2. Materials and Methods

In order to achieve an updated and extensive literature review on the theme of male breast cancer, an ample search was conducted between the 9 and 23 May 2022 in several international databases, as follows: (1) On www.scicencedirect.com, accessed on 9 May 2022, the search terms were: “breast cancer in male systematic review” between 2000 and 2022 and also “male breast cancer”, with the mention that “review” as type of article between 2018 and2022 and having “medicine and dentistry” as a domain. Furthermore, another association of terms that was searched for was “breast cancer in men systematic review”. (2) On www.pubmed.gov, the terms “male AND breast AND cancer” and also “breast cancer in men” were looked up with the settings: systematic reviews from the year 2000, in humans, articles in English. (3). Other quests on: www.pubmedncbi.nlm.nih.gov, www.oxfordjournals.org, and www.sciencedirect.com, accessed on 23 May 2022, and having as settings: reviews, in humans, article in English, domain medicine and dentistry, looked also for meta-analysis and randomized control trials, using the terms: ”male AND breast AND cancer” and “male breast cancer surgery”, and “male breast cancer (treatment) (systematic) review study”.

## 3. Results

### 3.1. Risk, Biology, Diagnosis

#### 3.1.1. General Facts and Specificities of the Geographical Distribution of Male Breast Cancer

Breast cancer tumorigenesis is a multi-step process that is believed to correlate with one or more distinct mutations in major regulatory genes at each step. To what extent would a multi-step progression model for sporadic breast cancer differ from that for hereditary breast cancer? This question was addressed by Kenemans [3]. The researcher found that hereditary breast cancer is characterized by breast cancer susceptibility based on a germline mutation in one allele of a high penetrance susceptibility gene (such as BRCA1, BRCA2, CHEK 2, TP53, or PTEN). Inactivation of the second allele of these tumor suppressor genes would be an early event in this oncogenic pathway (two-hit model of Knudson). Another idea presented by this study was that sporadic breast cancers are caused by a stepwise accumulation of acquired and uncorrected mutations in somatic genes, with no role played by germline mutations. Mutational activation of oncogenes, frequently coupled with non-mutational inactivation of tumor suppressor genes, is likely an early event in sporadic tumors, followed by at least four or five independent mutations in other genes.

Taking into consideration that men’s breast cancer causes, optimal treatments, and medical/psychosocial consequences are poorly understood, a systematic review of the literature in the English language was conducted by Ruddy [4] to identify various research materials relevant to breast cancer in men, between 1987 and 2012, materials that included at least 20 patients. Known risk factors encompass BRCA2 mutations, (80 times the risk of the general population, according to Fox [5], Peshkin [6]), and according to Nguyen [7], there are 11 other gene mutations, apart from BRCA, that might trigger a similar outcome. Other factors implicated are age, conditions that alter the estrogen/androgen ratio, and radiation. Due to the incomplete clinical picture resulting from insufficient studies, diagnostic and treatment tactics in men are generally induced from those in women, even though disease biology is different between the two sexes. According to a study by Gucalp [8], male breast cancer is almost exclusively hormone receptor positive (+), including androgen receptor (AR), and is associated with a higher prevalence of BRCA2 germline mutations, particularly in men at an increased risk for developing high-risk breast cancer. To better characterize male BC, additional research is required. Men may experience sexual and hormonal side effects of endocrine therapies, as well as unique psychosocial effects of the disease, as part of their survivor issues.

The CHEK2 kinase (Chk2 in mice) is a DNA damage response pathway component.

The impact of checkpoint kinase 2 *(CHEK2)* mutations as a prognostic factor in the pathogenesis of breast cancer was studied also by Ansari [9], who underlines that, in cell signaling pathways, *CHEK2* is regulated by the influence of upstream genes, and, that, in addition, *CHEK2* regulates a number of downstream genes. Moreover, mutations in *CHEK2* cause BC cells to be resistant to chemotherapy and expand to other organs. The research also mentions that the detection of mutations in *CHEK2* can be used as a prognostic factor for patient response to treatment and for targeting molecules involved in the proliferation of breast tumor cells that are downstream of *CHEK2*. Mutations such as c.1100delC and I157T distinguish susceptible patients to a metastatic form of the disease. 

In a systematic review by Liang [10], CHEK2*1100delC was associated with an increased risk of breast cancer in both men and women. In a study performed on mice and reported by Bahassi [11], it was found that subjects with CHEK2*1100delC SNP were predisposed to cancer with a strong gender bias. Furthermore, a recent systematic literature review by Chamseddine [12] looked at several male breast cancer (MBC) susceptibility genes. Different genes involved have been found, but the risk for individuals who have a pathogenic variant in each of these genes (i.e., penetrance) is not currently known, exactly. In order to better summarize current estimates of penetrance, an analysis of studies was done on the subject of reporting the penetrance of MBC susceptibility genes. From 12,182 abstracts, 15 studies measuring gene penetrance covering 5 putative male breast cancer genes were found: *ATM, BRCA1, BRCA2, CHEK2, and PALB2*. This study supports the conclusion that pathogenic variants in *ATM, BRCA2, CHEK2* c.1100delC, and *PALB2* increase the risk of MBC, while pathogenic variants in *BRCA1* may not be associated with an increased risk of MBC. Moreover, Friebel [13] shows in a systematic review that the cancer risk of women who have inherited a *BRCA1* or *BRCA2 (BRCA1/2)* mutation is highly variable.

Although additional research is necessary to confirm certain associations, sufficient information is available to use certain risk factors in risk counseling or lifestyle modification to reduce cancer risk in *BRCA1/2* mutation carriers.

A recent (2021) systematic review and meta-analysis by Davey [14] looks at the relevance of the 21-gene expression assay in male breast cancer. The 21-gene assay provides prognostic information for early female breast cancer patients with estrogen receptor positivity and human epidermal growth factor receptor-2 negativity (ER+/HER2−). This signature in male breast cancer has not been validated. The results of the research showed that, for early-stage, ER+/HER2− breast cancer patients undergoing 21-gene expression assay testing, the expected scores for females and males are comparable. In the absence of stage matching, these results must be interpreted with caution. Validation of the 21-gene MBC assay is still necessary in a future study with stage matching between the two sexes.

A study by Fentiman [15] underlines the endocrine risk factors. The significant increase in global age-standardized mean BMI in men is likely to lead to an increase in the incidence of diabetes of adulthood and metabolic bone disease (MBD).

Obesity is accompanied by metabolic changes that decrease androgens and sex hormone-binding globulin (SHBG), thereby increasing the availability of estrogens. Klinefelter’s syndrome (XXY) is associated with a 50-fold increase in MBC incidence compared to XY males; this is the strongest evidence for testicular dysfunction amplifying risk.

Symptomatically diagnosed cancers in men are typically advanced, indicating that earlier detection could improve prognoses. A research by Woods [16] identified potential screening benefits of screening high risk patients, such as high sensitivity and early detection.

In a review by Nofal [17], it was concluded that male breast cancer typically presents as a painless retro-areolar mass requiring triple evaluation. The diagnosis requires a high index of suspicion due to the lack of awareness of this type of cancer in males.

In a study dating from 2021, Pizzato [18] looked at mortality data in male breast cancer by analyzing, from 2000 to 2017, official death certification data and population estimates for breast cancer in men, reported by the WHO and Pan American Health Organization. Death rates standardized by age were computed for selected countries and regions worldwide. Between 2015 and 2017, central-eastern Europe had a rate of 2.85 per million people, and Russia had a rate of 2.22, placing them among the highest. North-western and southern Europe, the European Union as a whole, and the United States exhibited rates ranging from 1.5 to 2.0. Lower rates were observed in most Latin American nations, with values below 1.35/1,000,000, compared to 1.22/1,000,000 in Australia and 0.58/1,000,000 in Japan. Age-adjusted death rates decreased between 10 and 40 percent in 2000–2004 and 2015–2017 in north-western Europe, Russia, and the United States, and between 1.5 and 25 percent in the other regions studied, with the exception of Latin America (+0.8 percent). With the exception of central-eastern Europe, the anticipated rates for 2020 were favorable.

The favorable trends in male breast cancer mortality rates over the past several decades are likely primarily attributable to advances in management. In some areas, the higher mortality rate is due to delayed diagnosis and limited access to effective treatment.

Ndom [19] included in a literature review papers that contained data on both male and female breast cancers in Africa, and if both male and female breast cancer were available, the article was included. If two publications covered the same geographical region, only the one with the longer study period was included. In total, 1201 male and 36,172 female breast cancer patients from 27 African countries were analyzed using random effects models and meta-regressions with mixed effects. In addition, male breast cancer patients in Africa were diagnosed at an average age of 54.6 years, seven years older than female patients. Male breast cancers in Africa are characterized by their late onset, and the male-to-female breast cancer ratio in Africa is higher than in developed nations. Fentiman [15] emphasizes the fact that the higher rates of MBC in northern and equatorial Africa are largely attributable to liver damage caused by endemic bilharziasis and hepatitis B, which results in elevated estradiol (E2) levels from hepatic androgen conversion.

In a retrospective study on male breast cancer in Tunisia, Methamem [20] found that invasive ductal carcinoma was the most prevalent histological subtype (95 percent of our patients). The series was divided into three immunohistochemical groups, with luminal A (68.2%), followed by luminal B (27.3%), and a single patient with a triple-negative tumor (4.5 percent). At 5 and 10 years, the overall survival rate (OSR) was 83.2 percent and 76.8 percent, respectively. Recurrence-free survival (RFS) was 64.5 percent at 5 years and 58.6 percent at 10 years. Age, clinical and histological tumor size, the presence of distant metastases, and the occurrence of recurrence all had a significant impact on the OSR. Recurrence-free survival (RFS) was affected by age, clinical and histological tumor size, and dermal infiltration.

A study by Ssentongo [21] finds that regional, subregional, gender, and racial disparities influence breast cancer survival rates in Africa.

Consequently, measures are urgently required per region- and race-specific public health interventions coupled with prospective genetic studies to improve breast cancer survival in this region.

#### 3.1.2. Gynecomastia and Pediatric Cases

Gynecomastia has been described as the leading cause of male breast enlargement. This could be physiological, idiopathic, or pathological in nature, as stated in a review by Shaaban [22]. A review by Billa [23] found that 45 to 50 percent of adult men with GM may have an underlying pathology, such as aggravating medications, systemic diseases, obesity, endocrinopathies, or cancer. Mammography and ultrasound are both sensitive and specific for distinguishing GM from breast cancer. When clinical findings suggest malignancy and imaging results are inconclusive, histological confirmation should be sought. It is essential to distinguish between gynecomastia, a common cause of male breast enlargement, and breast cancer for proper treatment.

In comparison with core needle biopsy, fine-needle aspiration biopsy (FNA) has been demonstrated as sensitive and specific in assessing breast tumor lesions in female patients. Few studies of this nature have been conducted on men. In a study presented by Hoda [24], an evaluation was done of the patients who had fine-needle aspiration (FNA) at Massachusetts General Hospital. The procedure had been performed for palpable breast lesions, in the timeline January 2007–December 2016. The conclusion was that FNA allows for the evaluation and diagnosis of palpable male breast lesions in a sensitive, specific, and safe manner.

As presented in a study by Ghilli [25], secretory breast cancer (SBC) is one of the rarest forms of breast cancer (BC), accounting for the vast majority of BC in children. Nonetheless, it generates a great deal of interest due to its peculiar morphology and genetic characteristics. SBC is a rare form of breast cancer characterized by triple-negative characteristics and an unexpectedly favorable prognosis. More information is required to fully comprehend this cancer’s behavior, and genomic profiling may help improve its diagnosis and treatment.

Gynecomastia has been described also as appearing after a previous diagnosis of childhood cancer (of any type), as shown in a study by Shahriari [26], which shows that more than 80% of children with cancer today can be cured. The treatment of childhood cancer focuses not only on improving survival, but also on reducing late effects. The purpose is that children with a cancer diagnosis survive and enjoy a high quality of life. Gynecomastia and fertility outcomes of childhood cancer survivors should be considered in the follow-up of adolescents and young adults, and should be approached accurately and managed by multidisciplinary teams. Moreover, in a systematic review presented by Wang [27], subsequent male breast cancer (SMBC) in cancer survivors, compared to the general population can have an elevated risk of appearance, but the absolute risk is low. Male CCSs (childhood cancer survivors) with symptoms possibly related to SMBC should be thoroughly examined. 

#### 3.1.3. Metastases

A literature review by de Almeida Freire [28] draws attention towards uncommon metastatic sites. For instance, in the oral and maxillofacial region of male patients, breast metastases are exceedingly rare; however, clinicians should consider breast metastasis when evaluating reddish oral nodules in older patients, including men, particularly those with a history of malignancy. More so, in point of clinical approach, it is important to know and have in mind the aspect according to which, even if some metastatic sites are very rare, they can be the first clinical manifestation of an occult male breast cancer. This previous aspect was underlined by Kesting [29] and Gonzalez-Perez [30].

Invasive lobular carcinomas(ILC) are rarer than ductal forms and are known to have unusual metastatic locations, and they can appear as primary tumors, such as may be the case of the pancreas, for instance, as described by Mor [31].

Statistically, approximately 20% of cancer patients have brain metastases (BMs). This proportion rises with age to roughly 40 percent among those under 18 years old. However, the actual prevalence may be higher, as these estimates are typically restricted to individuals who are being evaluated for therapy. According to a study presented by Che [32], most BMs metastasize from lung cancer (40–50%), breast cancer (15–25%), and melanoma (5–20%). However, cancer patients with BMs continue to have a poor prognosis, with a relatively low median survival (2.9 months for newly diagnosed malignancies) and 2-year survival rate (8%). Increasing evidence suggests that gender is associated with the survivability of the vast majority of malignant tumors. In addition to that, many studies have demonstrated that the male gender is an independent risk factor for a shorter survival rate in BMs patients. The conclusion of the study was that middle-aged females had an increased risk of developing BMs, whereas middle-aged males with BMs had an increased risk of poor survival, an aspect underlined also by Leone [33] and Singh [34].

Although a few similar cases have been reported, there have been no reports of subtype conversion in similar cases. Consequently, Oh [35] presents the case of a male patient with brain metastasis of invasive ductal carcinoma and HER2 status conversion subsequent to metastasis.

Male breast cancer with brain metastasis is an extremely uncommon condition, with even rarer depiction at the level of the cerebellopontine angle, which can manifest with acute onset and rapid progression of symptoms: deterioration of the level of consciousness and intracranial hypertension, as explained by Tahrir [36] in a study describing a triple negative male breast cancer patient.

The period of time in which metastases to the brain can occur in relation to the moment of the initial diagnosis is emphasized by Furchinoue [37], who mentions a 24-year range.

#### 3.1.4. Image-Based Diagnostic Methods 

Due to the small size of male breasts, physiological and pathological processes arising from the breast and anterior chest wall may have similar clinical manifestations, as presented by Yang [38]. As described by Mango [39], men presenting with breast symptoms may pose unique diagnostic difficulties for the radiologist, especially if imaging findings are not typical for gynecomastia or carcinoma. When radiological findings are ambiguous or suspicious, imaging is frequently necessary to localize and characterize the lesion and guide biopsy. Mammography, digital breast tomosynthesis (DBT), and ultrasound are the cornerstones of breast imaging evaluation. Symptomatic male breast imaging begins with a diagnostic mammogram and targeted ultrasound in patients 25 years old, as reported in a review performed by Chesebro [40]. If the breast finding is insufficiently imaged or there is occult on mammography, targeted ultrasound is required.

Similarly, mammography must be performed if the breast finding on targeted ultrasound in a younger patient is suspicious. Occasionally, additional imaging techniques such as computed tomography (CT), magnetic resonance imaging (MRI), and positron-emission tomography (PET) can supplement the investigation and aid in the planning of treatment.

**Figure 1:**
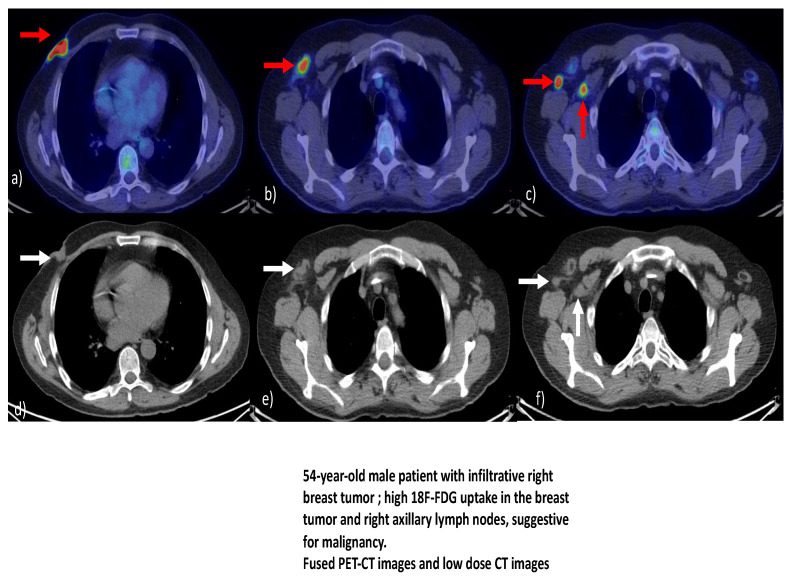
(**a**–**f**) Subsets illustrating PET-CT/ low dose CT aspects of male breast tumors. Courtesy of Dr. Mirela Gherghe, affiliated to the Nuclear Medicine Department of the Bucharest Oncology Institute and “Carol Davila” University of Medicine and Pharmacy, Bucharest, Romania.

In a systematic review by Dondi [41], which aimed to analyze the diagnostic performance and utility of 18F-FDG PET/CT in the evaluation of MBC, it was found that, despite the limitations of the review, 18F-FDG PET/CT appears to be an effective method for assessing MBC. The conclusion continued with the idea that further research is required to clarify the role of hybrid imaging with 18F-FDG in the evaluation of MBC, particularly in comparison to breast cancer in females. Figure 1a–f illustrates various aspects of PET-CT in a patient diagnosed with mail breast cancer.

In order to assess the potential added value of SPECT-CT quantitative analysis in the detection and differentiation of metastatic breast cancer lesions from degenerative lesions, a study was conducted by Gherghe [42]. The SUVmax value of metastatic bone lesions was significantly higher than that of degenerative bone lesions (*p* < 0.001). At an SUVmax cutoff value of 16.6 g/mL, the diagnostic accuracy of SPECT-CT quantitative data analysis revealed a sensitivity of 91.5 percent and a specificity of 93.3 percent. The conclusion of the research was that quantitative analysis of SPECT-CT data can improve the diagnostic accuracy of distinguishing metastatic bone lesions from degenerative bone lesions, leading to more appropriate treatment and improved follow-up in metastatic breast cancer patients.

In the given literature context that microcalcifications (MCs) are significant breast cancer disease markers, numerous studies have been conducted on their characterization in female breast cancer (FBC), but their composition in male breast cancer is unknown (MBC). According to Caldarone [43], as Raman spectroscopy (RS) is a molecular spectroscopy that can rapidly and without staining examine the biochemical composition of MCs, an algorithm to identify the mineral components present in MCs from Raman images can be used to study and compare MCs identified on breast cancer pieces from male to female patients. This suggests that these patients have characteristics that distinguish them from the FBC previously studied. 

In a review from 2018, Shin [44] found that the literature on the use of breast MRI in male patients is also extremely limited.

Although it is uncommon and not recommended as a standard clinical practice to perform breast MRI on male patients, even in the presence of a breast cancer diagnosis, there are a few instances in which MRI may be helpful to clinicians and surgeons.

#### 3.1.5. Pathology

Similar to the “female breast”, the “male breast” is home to a number of pathological conditions. However, histological-anatomical differences in the female breast result in numerous variations in disease frequency and presentation, as explained by Onder [45]. We hereby present in this section general information on the common types found and an exhaustive list of uncommon types versus their prognosis and clinical significance.

Shaaban [22] finds that the most prevalent histology is grade 2 ductal carcinoma with no special subtype. MBC is frequently of the luminal A phenotype comparable to postmenopausal breast cancer in women. A study by Fentiman [46] showed that using hierarchical clustering, ER was clustered with PR in FBC, but with ER and AR in MBC.

Oncotype DX appears to be effective in determining recurrence risk in selected MBC based on limited data. A study by Cho [47] looked at the use of HE images, a deep-learning algorithm that may be able to predict the efficacy of adjuvant chemotherapy in cancer patients. The Lunit SCOPE algorithm was developed using HE slides from 1343 breast cancer patients. Lunit SCOPE was trained using the 21-gene assay (Oncotype DX) and histological parameters to predict recurrence. The risk prediction model accurately predicted the Oncotype DX score > 25 and the recurrence survival of the validation cohort and TCGA cohorts. The predicted risk was positively associated with proliferation-associated Oncotype DX genes and negatively correlated with estrogen-related prognostic genes. The risk of cancer recurrence and the early-stage hormone receptor-positive breast cancer patients who would benefit from adjuvant chemotherapy were predicted by an integrative analysis utilizing Lunit SCOPE.

Papillary in situ and invasive carcinomas are not uncommon in the male breast, unlike the female breast. Zhong [48] finds in a review that papillary lesions of the male breast papillary carcinomas span a wide clinicopathological spectrum, and both invasive and noninvasive papillary carcinomas have a favorable prognosis, as it is also reported by Avau [49]. 

As presented by Fox [5], metastases to the breast also occur, but according to Akinseye [50], they are rare and a differential diagnosis with a primary lesion should be done. Lymphoma or leukemia, lung cancer, melanoma, prostate, gastric, renal, endometrial, pancreatic, esophageal, and thyroid cancers are the most prevalent primary malignancies affecting the breasts (in descending frequency). In most cases, the presence of breast metastases indicates a widespread disease. The prognosis is typically poor, and treatment focuses on the primary cancer.

For instance, Anagnostopoulou [51] describes how male breast lymphoma is a rare extranodal lymphoma of the mammary gland that may be primary or secondary. A breast lesion’s excisional biopsy revealed chronic lymphocytic leukemia (CLL) with plasmacytoid features and immunoglobulin Gkappa monotypic expression in the mammary tissue.

Atypical ductal hyperplasia (ADH) greatly increases a woman’s risk of developing breast cancer. Nevertheless, not much is known about the effects of ADH in men. In a study conducted by Coopey [52] with a mean follow-up period of 6 years, no males in the series developed breast cancer. The data inferred that, either ADH in men does not have the same risk as ADH in women, or surgical excision of a symptomatic gynecomastia lesion in men can effectively reduce the odds of breast cancer.

As presented by Wu [53], male cases of accessory breast cancer and sweat gland cancer associated with extramammary axillary Paget’s disease are uncommon. In clinical diagnosis and treatment, it is necessary to precisely identify the disease and devise an appropriate treatment plan based on the patient’s condition. 

Mesenchymal lesions of the breast include benign, reactive, and malignant conditions, as described by Carder [54]. They typically present to the pathologist as spindle cell lesions, and many have distinctive histological and immunohistochemical characteristics, an aspect emphasized also by Raj [55].

A phyllodes tumor (PT) is a prototypical fibroepithelial neoplasm that accounts for 1% of breast neoplastic lesions that are typically detected in females and rarely in males. On the basis of predetermined morphological criteria, the World Health Organization classifies tumors as benign, borderline, or malignant. Infrequently documented in the English literature, squamous differentiation in phyllodes tumors represents epithelial metaplasia, as shown also by Panigrahi [56]. It has been difficult to determine which phyllodes tumors may behave aggressively. In this context, a study described by Lerwill [57] looks at the discovery of MED12 mutations in the pathogenesis of fibroepithelial tumors, along with other gene abnormalities in the progression pathway, which has allowed for the improvement of prognosis and diagnosis. Another study, by Ma [58], finds that nonbenign PTs can be predicted independently by tumors in the family, lobulation, and cystic components. Moreover, the prediction nomogram developed based on these characteristics can be used as a supplementary instrument for preoperatively classifying PTs.

Dermatofibrosarcoma protuberans (DFSP) is a soft tissue sarcoma that accounts for approximately 1 percent of all tumors. In addition, DFSP is frequently observed on the trunk and extremities, whereas breast occurrences are uncommon, as stated by Bouhani [59]. A high index of clinical suspicion is required for its detection and differentiation from simple wound complications and local recurrences of other benign lesions, as is shown by Sung [60].

Paget’s disease in the breast manifests with eczematous changes of the nipple-areolar complex and, in the majority of cases, is accompanied by an underlying in situ or invasive breast carcinoma, an aspect underlined by Vergine [61].

Paget’s disease is characterized histologically by epithelial cells with an abundance of basophilic or amphophilic, finely granular cytoplasm, and a large, centrally located nucleus, which are most prevalent in the lower epidermal layers. Due to the rarity of the condition among breast cancers and the rarity of breast cancer in men, knowledge of the disease’s presentation, course, and optimal treatment in male patients is derived primarily from case reports and extrapolation of findings from studies in female patients. Paget’s disease must be distinguished from eczema, Bowen’s disease, squamous cell carcinoma, and melanoma, among others. In a study by Adams [62], it was also emphasized that Paget’s disease must be identified clinically and pathologically, as the superficial lesion may be the only indication of an underlying ductal carcinoma and its presence may have prognostic significance.

**Figure 2:**
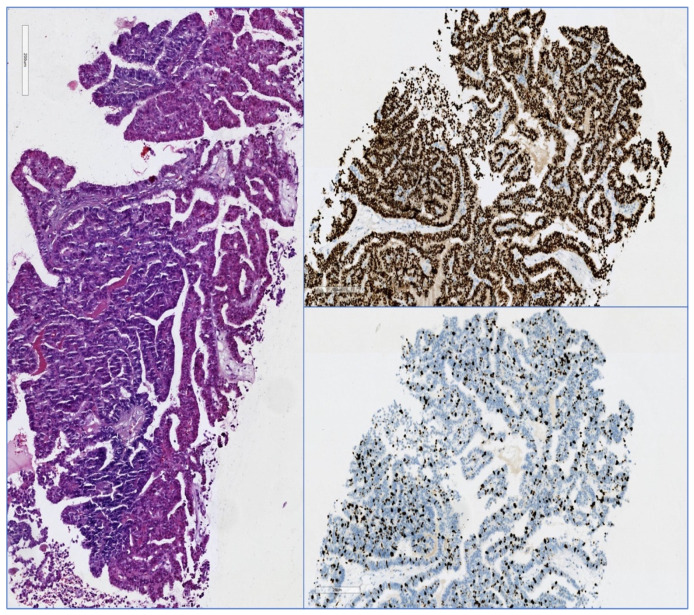
Biopsy sample from a breast tumor of a male 66 y.o.: left panel: papillary carcinoma of the breast, HE, 100×, top right panel: estrogen receptor positive in tumor cells nuclei (Allred score: 8), bottom right panel: Ki-67 positive in ~75% of the tumor cells nuclei, IHC, 100×.

**Figure 3:**
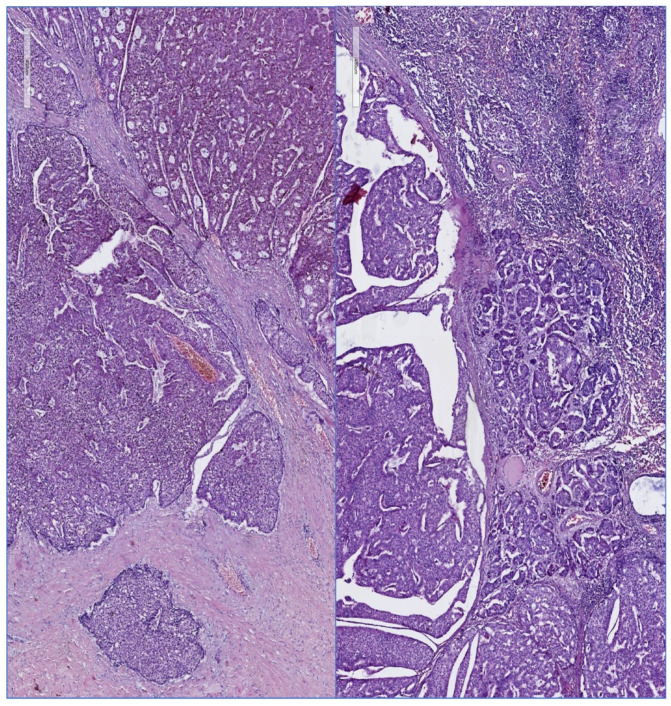
Surgical resected specimen, same case: left panel: invasive mammary carcinoma, right panel: tumor invasion in an axillary lymph node, HE, 100×. Figure 2 and Figure 3 are presented courtesy of Dr. Mihai Ceausu, affiliated to the Pathology department of the “Prof. Dr. Al. Trestioreanu” Bucharest Oncology Institute and Associate Professor at the “Carol Davila” University of Medicine and Pharmacy, Bucharest, Romania.

Figure 2 and Figure 3 illustrate different pathology caracteristics of breast cancer in male patients.

### 3.2. Treatment 

Tamoxifen is a selective estrogen receptor modulator (SERM) that is utilized to treat all stages of hormone receptor-positive breast cancer in men and women. Aromatase inhibitors (AIs) are a class of drugs used to treat postmenopausal women and men with breast cancer. Aromatase catalyzes an essential aromatization step in the synthesis of estrogen.

In order to determine the effect of adjuvant treatment with tamoxifen and aromatase inhibitors (AI) on the survival of male breast cancer patients, a study by Eggemann [63] analyzed 257 male breast cancer patients with positive hormone receptor status. The overall survival of male breast cancer patients treated adjuvantly with tamoxifen was significantly greater than that of those treated with an aromatase inhibitor.

According to the ASCO guideline published in 2020, as presented by Hassett [64], many of the treatments for breast cancer in men are similar to those used for women. Men presenting hormone receptor-positive breast cancer who are candidates for adjuvant endocrine therapy have an indication to receive tamoxifen for an initial duration of five years; those who cannot be given tamoxifen due to counterindications may be prescribed a gonadotropin-releasing hormone agonist/antagonist plus aromatase inhibitor. After five years of tamoxifen therapy, those having tolerated the treatment and continuing to have high odds of recurrence may be given an additional five years of treatment. Men with early-stage disease should not be administered bone-modifying agents to prevent recurrence, but these substances may still be administered to prevent or treat osteoporosis. With the exception of cases of visceral crisis or rapidly progressive disease, men with advanced or metastatic disease should receive endocrine therapy as the initial treatment option. Targeted systemic therapy may be used to treat advanced or metastatic cancer using the same indications and combination treatments available for women. Men with a history of breast cancer treated with lumpectomy should receive an ipsilateral annual mammogram regardless of genetic predisposition; men with a history of breast cancer and a genetic predisposition mutation may receive a contralateral annual mammogram. All men diagnosed with breast cancer should receive genetic counseling and testing for cancer susceptibility genes in the germline.

In a study done by Trapani [65] in 2021 on the global aspect of treatment standards in breast cancer, it was found that this global landscape of BC treatment standards reveals that the majority are not context-appropriate. Research on the formulation of cancer treatment standards and novel platforms for developing and disseminating resource-appropriate guidance are of the utmost importance.

T extent of changes in estradiol levels in male patients receiving standard endocrine therapies for hormone receptor-positive breast cancer is unknown and the impact of these changes on sexual function and quality of life has not been adequately evaluated, as shown in a randomized clinical trial conducted by Reinisch [66]. In order to complete the research, patients were randomly assigned to 1 of 3 treatment arms for 6 months: tamoxifen alone, tamoxifen plus gonadotropin-releasing hormone analogue (GnRHa), or aromatase inhibitor (AI) plus GnRHa. The primary outcome parameter was represented by the change in estradiol concentrations from baseline to three months. After 3 and 6 months, secondary endpoints included changes in estradiol levels, additional hormonal parameters, adverse effects, sexual function, and quality of life. This phase 2 randomized clinical trial revealed that AI or tamoxifen plus GnRHa versus tamoxifen alone resulted in a sustained reduction in estradiol levels. The decreased hormonal parameters were associated with diminished sexual performance and life quality. 

Giving an expert opinion on best methods of approach, Duso [67] presents that there is a significant medical need to include male breast cancer patients in (more) prospective clinical trials. The call for equality in breast cancer care can be pursued in two divergent ways: (i) a gender-neutral delivery of breast cancer information, and (ii) the creation of separate sections in common studies, one for the more prevalent female breast cancer and the second for the rare male breast cancer. We propose to differentiate male breast cancer care, recognizing that males have distinct onco-sexual and social needs that can only be shared partially with women.

In a review by Ahmed Jang Khan [68], it was found that, in certain cases, breast conserving surgery (BCS) with sentinel lymph node biopsy (SLNB) remains an alternative to mastectomy for men with early-stage breast cancer. The identification and false-negative rates for SLNB were comparable to those of breast cancer in female patients, according to a study presented by Lin [69], who also shows that survival is improved by post-mastectomy radiation to the chest wall and 5-year tamoxifen treatment.

Disease staging and sentinel lymph node biopsy were also studied by Carter [70], Gherghe [71], and Bordea [72], who show that standard treatment for women with clinically N0 breast cancer is sentinel lymph node biopsy (SLNB) and that it can also be associated, during the same intervention, with the localization of an occult breast lesion. However, there are no randomized controlled trials determining the optimal surgical management of the axilla in men. In males with clinically N0 breast cancer, the use of SLNB alone has increased while ALND has declined. Patients who underwent SLNB alone during the later time period, as shown by Carter [70], however, exhibited worse clinical characteristics and experienced variations in adjuvant therapy. This indicates a growing acceptance of SLNB for axillary management. Methods of axillary staging and their influence on the prognosis of males with breast cancer warrant additional investigation.

#### 3.2.1. Breast Conserving Surgery versus Mastectomy 

Fentiman [73] underlined that it is possible to make a compelling case for the use of neoadjuvant endocrine therapy to facilitate breast-conserving surgery. Although nomograms for predicting nodal status are inadequately calibrated, sentinel node biopsies have been utilized successfully to stage MBC. Male mastectomy is associated with psychological side effects, and there is no evidence that the needs of those with MBC are being met. The conclusions drawn by the previously mentioned research was that collaborative research is required so that men can participate in meaningful randomized controlled trials (RCTs) to provide a rational, evidence-based basis for the surgical treatment of MBC. Furthermore, Williams [74] discusses the context for neoadjuvant treatment in relation to advanced disease and aims at the determination of the prevalence of neoadjuvant therapy (NT) in MBC patients and its effect on BCT (breast conserving therapy). The conclusion was that males with invasive breast cancer are expected to have a low BCT rate, but NT appears to decrease the use of mastectomy in patients with locally advanced cancers. Understanding the effects of BCT on locoregional recurrence, disease-free survival, and overall survival for MBC requires additional research.

In a study by Bakalov [75], it was underlined that, while adj-RT after BCS is associated with decreased mortality in MBC patients, adj-RT is omitted in up to one-third of MBC cases after BCS, despite being the standard of care.

Breast conserving surgery (BCS) was studied in comparison with mastectomy in male breast cancer (MBC). In a study by Saunder [76], a systematic literature review was done on studies that reported one or more of the following: overall survival (OS), disease free survival (DFS) and disease specific survival (DSS) stratified (sorted, selected) by surgical treatment (BCS and/or mastectomy), and/or radiotherapy compliance with BCS. The conclusion of the analysis was that the majority of studies found no differences between BCS and mastectomy in which concerns the DFS, DSS, or OS. The results emphasized that BCS is a viable treatment option for MBC because it was associated with comparable oncologic outcomes to mastectomy, as was also shown by de La Cruz [77]. However, the low rates of radiotherapy adherence among male patients who underwent BCS are concerning and demonstrate the importance of involving patients with MBC in the selection of a surgical treatment strategy.

A study by Deldar [78] looks at the aspects of postmastectomy reconstruction in male breast cancer and concludes that there is limited availability of research on chest reconstruction after mastectomy in male breast cancer patients.

Nonetheless, the available evidence suggests that reconstruction can restore a patient’s body image; therefore, it should be considered and discussed routinely with male patients.

#### 3.2.2. Adjuvant Treatment

Tamoxifen is the only endocrine agent approved for the prevention and treatment of premenopausal and postmenopausal estrogen-receptor-positive breast cancer, as well as the treatment of male breast cancer.

Endoxifen, a secondary metabolite resulting from CYP2D6-dependent biotransformation of the primary tamoxifen metabolite, N-desmethyltamoxifen (NDT), is a more potent antiestrogen than either NDT or tamoxifen, the parent drug, as indicated by Dreger [79], Sanchez-Spitman [80], and Ahmed [81]. In addition to its effects on ER, endoxifen’s antitumor effects may involve additional molecular mechanisms of action. In phase 1/2 clinical studies, as is presented by Jayaraman [82], the efficacy of Z-endoxifen, the active isomer of endoxifen, was evaluated in patients with endocrine-refractory metastatic breast cancer, as well as patients with gynecologic, desmoid, and hormone-receptor-positive solid tumors, and demonstrated promising antitumor activity.

Palbociclib is a targeted or biological therapy drug which belongs to the CDK (cyclin dependent kinase) inhibitors and can be used in locally advanced and secondary breast cancer that is estrogen-receptor positive and HER2negative.

A report by Kraus [83] investigated the advantages and disadvantages of palbociclib plus endocrine therapy (ET) in men with hormone receptor-positive (HR+)/human epidermal growth factor receptor 2 negative (HER2) metastatic breast cancer (MBC).

A review of the global safety database revealed no new safety signals in men treated with palbociclib. Real-world data indicated that palbociclib plus ET is beneficial for men with MBC, with a safety profile consistent with previous findings in women with MBC.

This report’s data on palbociclib in women and men, including clinical trial data, real-world data, and a well-established risk/benefit profile, led to US approval of an indication expansion for palbociclib to include men with metastatic breast cancer.

Ribociclib belongs to the class of drugs known as kinase inhibitors. It functions by inhibiting the action of an abnormal protein that instructs cancer cells to proliferate.

Leuprolide is a GnRH agonist approved by the FDA for the treatment of endometriosis, uterine leiomyomata (also known as uterine fibroids), central precocious puberty in children, and advanced prostate cancer. Off-label uses include, among others, the treatment of breast cancer and hormone therapy for male-to-female transgender patients.

Goserelin is a drug used to suppress the production of sex hormones (testosterone and estrogen), specifically gonadotropin-releasing hormone agonist (GnRH agonist).

As presented recently by Campone [84], CompLEEment-1 (NCT02941926) is a single-arm, open-label, multicenter phase IIIb study evaluating the safety and efficacy of ribociclib plus letrozole (RIB + LET) in a large, diverse cohort of patients who have not previously received endocrine therapy (ET) for advanced disease, in an intent to provide treatment alternatives for advanced male breast cancer HR+, HER2−. Methods: Patients with hormone receptor-positive (HR+), human epidermal growth factor receptor 2-negative (HER2−) advanced breast cancer (ABC) who had no prior chemoradiotherapy (ET) and 1 prior line of chemotherapy for advanced disease were administered RIB + LET. Also administered to male patients was goserelin or leuprolide. Safety and tolerability were the primary endpoint; efficacy was a secondary endpoint. Males exhibited the same clinical benefit and overall response rates as the entire population. This study supports the use of RIB + LET in male patients with HR+, HER2− ABC.

### 3.3. Breast Cancer in Transgender Patients

As summarized in a research by T’Sjoen [85], The Endocrine Society recommends estrogens in conjunction with androgen-blocking drugs for transgender women. Feminizing treatment with estrogens and antiandrogens results in desirable physical changes, including increased breast growth, decreased facial and body hair growth, and fat redistribution in a female pattern. Patients should be informed of potential adverse effects, particularly those at risk for venous thromboembolism. 

The Endocrine Society’s recommendations for transgender men include testosterone therapy for virilization, voice deepening, cessation of menstruation, and increases in muscle mass and facial and body hair. Due to the lack of evidence, gender nonbinary individuals should be treated on an individualized basis. Teenagers may be administered a pubertal suspension containing GnRH analogs, followed by sex steroids. Before any hormonal intervention, options for fertility preservation must be discussed. Morbidity and cardiovascular risk are unaffected by cross-sex hormones in transgender men, whereas it is unknown in transgender women.

Cancers caused by sexual steroid use are possible, but uncommon. The term “transgender” refers to people whose gender identity and/or gender expression is different from the sex they were assigned at birth. As the number of individuals undergoing gender-affirming hormone therapy and gender-affirming surgery increases, radiologists must know progressively more about this population in order to be able to attend them properly. Even if diagnostic imaging methods and approaches for transgender individuals are similar to those for cisgender women, screening guidelines are different. Several professional and institutional guidelines have been developed to address breast cancer screening in the transgender population, particularly mammography screening in transfeminine individuals undergoing hormone therapy, as emphasized by Parikh [86].

#### 3.3.1. Male to Female

The therapeutic transition from male to female usually begins in late adolescence or adulthood, with the average onset being around 30 years of age. Current hormonal treatment protocols in transgender women combine high doses of estrogen and antiandrogen treatment to reduce testosterone levels in the blood. Likewise, the concomitant administration of progesterone would reduce the potential risk of breast cancer and cardiovascular events, as described by Martinez Ramos [87]. Male-to-female (MtF) breast cancer cases have nevertheless been reported since 1968, but the breast cancer risk of MtF patients remained unknown at the moment in which Hartley [88] decided to study the subject by looking at literature updates. Among the conclusions, there was the fact that breast cancer is present in MtF patients, who typically present with a palpable mass at a younger age. Furthermore, pathology-confirmed breast implant-associated anaplastic large cell lymphoma was described in the context of breast augmentation with bilateral silicone implants, as reported by Ali [89]. 

#### 3.3.2. Female to Male

The breast cancer risk and screening recommendations for transgender men or female-to-male (FtM) patients are still unknown, according to a systematic review presented by Hartley [90] that looked at patient demographics, breast cancer characteristics, presentation, and treatment. The conclusion was that breast cancer is present in transgender men, with risk dependent on top surgery; those who have had top surgery appear to have a lower risk than natal females.

Female-to-male (FtM) transsexuals, in the context of their testosterone therapy for masculinization, can have a modified risk of developing breast cancer. The purpose of the study by Fledderus [91] was to examine the evidence regarding the risk of testosterone therapy on breast cancer in female-to-male transsexuals and to assess breast cancer screening in this subgroup. The research concluded that few cases of FtM transsexuals with breast cancer have been documented. However, cases such as these alert physicians to the possibility that FtM transsexuals may develop breast cancer. Radiological screening of FtM transsexuals for breast cancer prior to mastectomy and histological screening of the mammalian tissue after mastectomy should be taken into account; physicians should appreciate and further decide with each individual FtM transsexual if screening is imposed by clinical or paraclinical data.

An analysis from 2020 by Patel [92], looking at the long-term effect of hormone replacement therapy HRT, found that, due to the paucity of long-term studies tracking breast pathology among transgender men and women, information about the risks associated with HRT is limited and often contradictory (in the current literature and in this setting). The study concluded that the long-term effects of off-label pharmaceutical use to modify hormone levels and sexual characteristics in transgender patients have not been adequately studied. The propensity of steroid hormones to promote the development of certain cancers raises concerns regarding the safety of varying drug doses and combinations. Additional clinical and laboratory research is required to better establish dosing and safety guidelines for transgender patients.

### 3.4. Second Cancers Associated with Breast Cancer in Men

According to the definition of the National Cancer Institute, “second primary cancer” is a term used to describe a new primary cancer in a person who has had cancer in the past. Second primary cancers may develop months or years after the primary cancer has been diagnosed and treated. Certain cancer treatments, such as chemotherapy and radiation therapy, may increase the likelihood of developing a second primary cancer. Certain inherited gene mutations (changes) and exposure to certain cancer-causing substances, such as tobacco smoke, may also increase the risk of developing a second primary cancer.

Decades of research have been devoted to the risk of second cancers among breast cancer patients. Men’s second primary tumors, in contrast with those found in women, are poorly understood. Men’s breast cancer risk factors, such as genetic, hormonal, and environmental factors, parallel the causes of breast cancer in women. A review by Grenader [93] examines the literature concerning the risk of developing a second cancer in male breast cancer patients. Patients with a history of male breast cancer are more likely to develop a second ipsilateral or contralateral breast tumor (standardized incidence ratio 30–110), a phenomenon also underlined by O’Leary [94]. The risk of subsequent contralateral breast cancer was greatest in men younger than 50 years old at the moment of the initial diagnosis of the neoplasm. Diverse information is available on second primary cancers besides breast cancer. According to one study, the incidence of cancers of the small intestine, prostate, rectum, and pancreas, as well as non-melanoma skin cancer and myeloid leukemia, has increased. Other researchers did not find an increase in the overall risk of subsequent cancer development among men initially diagnosed with primary breast cancer. Although sarcoma, lung, and esophageal cancers are well-known complications of radiation therapy for breast cancer in women, there is no evidence that these cancers are associated with radiation therapy for breast cancer in men.

Cancer treatment is an especially trying time for the patients. They frequently experience multiple side effects concurrently, resulting in a decline in health-related quality of life (HRQoL). A study presented by Charalambous [95] provides evidence regarding the co-occurrence and interrelationships of pain, anxiety, depression, and fatigue in breast and prostate cancer patients. This research provides evidence that targeting fatigue, anxiety, and depression may have a meaningful effect on pain as a related symptom and may have a positive impact on the HRQL of breast and prostate cancer patients.

### 3.5. Prognosis

In an update on research concerning male breast cancer, Benassai [96] and Malinda [97] state that the outcome of the MBC is worse than the outcome of FBC.

In a comparison between prognostic factors studied in both sexes, Yao [98] found that as opposed to FBC patients, MBC patients were discovered at more advanced TNM stages, with higher tumor grades, and with a greater proportion of hormone receptor-positive tumors. In addition, the locations of breast tumors differed significantly between males and females, and longer survival rates were found in women. Age, race, TNM stages, tumor grades, estrogen receptor (ER)/progesterone receptor (PR), and human epidermal growth factor receptor-2 (HER-2) overexpression were found to be independent prognostic factors in female breast cancer, according to multivariate Cox regression and competing risks analyses.

MBC and FBC patients had the same risk factors, but PR and HER-2 status did not have an independent influence on survival (all *p* > 0.05).

Further conclusions were that, in the coming years, it is reasonable to anticipate an increase in the sensitivity of multigenic tests, allowing for a more accurate prediction of recurrence risk. This could result in substantial changes in the selection and duration of treatment, with surprising outcomes.

Treatment adherence is a very important factor in which concerns prognosis, and a study by Ali [99] compared the endocrine therapy adherence, discontinuation, and survival outcomes of male and female breast cancer patients using the SEER-Medicare linked database. The primary endpoints were rates of endocrine therapy adherence and discontinuation (ET). Adherence was defined as a gap between Medicare prescriptions of less than 90 days. A discontinuation was defined as a 12-month interval between Medicare prescriptions or longer. Secondary outcome measure was the association between ET use and overall survival (OS). Men were significantly more loyal than women, but there was no difference between the sexes in terms of abandonment. On ET, both men and women exhibited a significant survival improvement.

Pensabene [100] describes that, in addition to non-genetic risk factors, genetic alterations, such as pathogenetic variants in *BRCA1/2* and other moderate-/low-penetrance genes, have been identified as pathogenic factors for MBC. The detection of alterations in predisposing genes, especially *BRCA1/2*, and the identification of oncogenic drivers distinct from FBC may have preventative and therapeutic implications. However, the approved treatments for MBC are identical to those for FBC. Cancer genetic counseling must be considered in the diagnostic workup of MBC, regardless of the presence or absence of an oncological family history.

The goal of a study by Stahl [101] was to analyze men with de novo stage IV breast cancer and known estrogen receptor (ER) and progesterone receptor (PR) statuses who underwent systemic therapy, with or without surgery. Patients who died in the first six months were excluded from the study. In male patients with de novo stage IV breast cancer who were ER+ or PR+, it was discovered that those who received surgery, radiation therapy, and systemic therapy (trimodality) had a significant survival advantage over those who received only systemic therapy. The data also revealed a downward trend in the use of surgery in this cohort over time.

In a study based on the proteomic profiling of male breast cancer, Zografos [102] found a total of 2352 proteins, corresponding to 1249 single gene products with diverse biological functions. A panel of 119 differentially expressed tissue proteins was identified in MBC samples versus controls; 90 were found to be over-expressed in MBC tissues and 29 were found to be downregulated. Concurrently, 844 proteins were detected only in MBC tumors, whereas 197 proteins were expressed exclusively in mammary samples from healthy controls. Differential proteomic expression was identified in MBC tissue, resulting in a better understanding of MBC pathology and highlighting the need for individualized care for male patients.

### 3.6. Future Trends and Potential Therapeutic Targets in Breast Cancer

#### 3.6.1. Aquaporins

Aquaporins (AQPs) are membrane channels that belong to the large family of major intrinsic proteins (MIPs), of which there are thirteen classes with tissue-specific distributions in humans, as summed up by Khan [103] and Magouliotis [104]. As important physiological modulators of water and solute homeostasis, mutations and dysfunctions in aquaporins have been linked to pathologies in all major organs. Jung [105] emphasizes the fact that an anomalous expression of AQPs has been observed in numerous types of cancer cells and cancer stem-like cells, and it has been proposed as a marker for the proliferation and progression of cancer cells. Consequently, a more comprehensive understanding of AQPs could increase interest in the cell stemness accompanied by AQP expression. The traditional view of AQPs’ role in therapeutic plans restricted them and their regulators to managing a limited range of diseases, such as diabetes insipidus and syndrome of inappropriate ADH secretion. However, additional research, particularly in the third millennium, for instance a study by Ala [106], has revealed that their cooperation in water transmission control can be manipulated to address other burden-imposing diseases, including cirrhosis, heart failure, Meniere’s disease, cancer, bullous pemphigoid, eczema, and Sjogren’s syndrome. Khan [103] shows the important association between breast cancer and aquaporins, underlining that AQPs 1, 3, and 5 are highly expressed in breast, endometrial, and ovarian cancers, consistent with their gene regulation by estrogen response elements. Bystrup [107] considers that understanding the underlying molecular mechanisms of how AQP5 contributes to cancer development and progression is crucial for the potential use of AQP5 as a prognostic biomarker and for the development of targeted intervention strategies for the treatment of breast cancer patients. A similar observation was presented by Traberg-Nyborg [108] on aquaporin-1 and by Milkovic [109] on aquaporins 3 and 5. In addition to the above-mentioned statement, a study by Moosavi [110] found that overexpression of AQP1, AQP3, and AQP5 is inextricably linked to carcinogenesis, metastasis, decreased survival rate, lymph node metastasis, a worse prognosis, and cellular migration and that furthermore, cancer therapies associated with these markers suggest AQP decreases during treatment. A study by Zhu [111] that analyzed the mRNA of AQPs indicated that high AQP0, AQP1, AQP2, AQP4, AQP6, AQP8, AQP10, and AQP11 mRNA expression levels were significantly associated with improved relapse-free survival (RFS) and that, in contrast, AQP3 and AQP9 were associated with a shorter RFS in breast cancer patients, indicating that these two genes may serve as targets for future chemotherapy.

#### 3.6.2. The Androgen Receptor

Forooshani [112] mentions that, unfortunately, the prognosis for patients with hormone-negative tumors or patients with therapy-resistance and metastasis remains dismal. New biomarkers are urgently required to predict the disease’s progression, make better therapy decisions, and increase patient survival. In this regard, the Androgen Receptor (AR), a member of the superfamily of nuclear hormone receptors along with ER and PgR, emerges as an intriguing characteristic widely expressed in human BCs. The precise tumorigenic mechanism of the androgen receptor (AR) and the role of its endogenous ligands are not yet well understood, despite recent advances.

Yardley [113] considers that efforts to validate the AR as a therapeutic target should concentrate on identifying new markers predictive of sensitivity to AR-targeted drugs.

#### 3.6.3. Breast Pre-Cancer Atlas

Microenvironmental and molecular factors mediating the progression of Breast Ductal Carcinoma in situ (DCIS) are poorly understood, which hinders the development of prevention strategies and the testing of treatment de-escalation in a safe manner.

A study by Nachmanson [114] addressed methodological limitations and characterized the mutational, transcriptional, histological, and microenvironmental landscape of 85 multiple micro-dissected regions from 39 cases.

Phenotypic and subtype heterogeneity was frequently associated with underlying genetic heterogeneity, and according to the inferred phylogeny, regions with low-risk characteristics preceded those with high-risk characteristics.

The spatial analysis of B- and T-lymphocytes revealed three immune states, including an epithelial excluded state located preferentially in DCIS regions and characterized by the histological and molecular characteristics of immune evasion, independent of molecular subtypes.

This breast pre-cancer atlas with its unique integration of observations will aid in the planning of future expansion studies and the development of more accurate models of outcomes and progression risk.

Lactate dehydrogenase C (LDHC), an anticancer target with tumor-specific expression and immunogenicity, is a cancer testis antigen (CTA). Analysis of breast cancer patient cohorts from The Cancer Genome Atlas (TCGA) and Molecular Taxonomy of Breast Cancer International Consortium (METABRIC) indicates that upregulation of LDHC expression is associated with a poor prognosis.

A study by Naik [115] examined whether LDHC is involved in regulating genomic stability and whether it could be targeted to influence the cellular fitness of tumor cells.

In four breast cancer cell lines, silencing LDHC significantly increased the number of giant cells, nuclear abnormalities, DNA damage, and apoptosis. Cells depleted of LDHC exhibited aberrant cell cycle progression accompanied by differential expression of cell cycle checkpoint and DNA damage response regulators. This previously mentioned research demonstrates the therapeutic potential of targeting LDHC to reduce cancer cell survival and enhance sensitivity to agents that cause DNA damage or inhibit its repair.

#### 3.6.4. Alternative Splicing

Alternative splicing allows cells to diversify their proteome in order to carry out complex biological functions and respond to external and internal stimuli. The spliceosome is the multiprotein-RNA complex responsible for the complex process of alternative splicing.

As a result of abnormal spliceosomes or splicing factors, aberrant splicing can drive the development and progression of cancer, as shown in a review by Murphy [116].

Recent mapping of the spliceosome, its associated splicing factors, and their relationship to cancer has paved the way for novel therapeutic approaches that capitalize on alternative splicing’s widespread influence.

#### 3.6.5. Squalene Epoxidase

Over fifty percent of cancer patients are treated with radiotherapy; however, radiotherapy as a monotherapy is frequently insufficient and requires a nontoxic radiosensitizer.

Squalene epoxidase (SQLE) regulates the biosynthesis of cholesterol by converting squalene to 2,3-oxidosqualene.

A study by Hong [117] investigated the significance of SQLE in breast cancer and non-small cell lung cancer (NSCLC), two cancers that are frequently treated with radiotherapy.

SQLE-positive IHC staining was observed in 68% of breast cancer and 56% of NSCLC specimens, compared to 15% and 25%, respectively, in normal breast and lung tissue.

Significantly, SQLE expression was an independent predictor of a poor prognosis, and pharmacologic inhibition of SQLE increased the radiosensitivity of breast and lung cancer cells. The conclusion of the research was that squalene epoxidase inhibitors are novel tumor-specific radiosensitizers that promote ER stress and suppress homologous recombination, providing a novel potential therapeutic approach to improve radiotherapy efficacy.

#### 3.6.6. The Unfolded Protein Response

Estrogen receptor (ER) is a therapeutic target for patients with ER-positive breast cancer.

In endocrine-resistant breast cancer, paradoxically, this is also the initial site where estrogen (E2) induces apoptosis.

How ER displays distinct functions in various contexts is the subject of numerous studies. Among those, a study by Fan [118] found that unfolded protein response (UPR) is closely correlated with ER-positive breast cancer, according to compelling evidence.

Treatment with antiestrogens induces a mild UPR through ER, activating three UPR sensors in the endoplasmic reticulum: PRK-like endoplasmic reticulum kinase (PERK), inositol-requiring enzyme 1 (IRE1), and activating transcription factor 6 (ATF6).

These sensors then interact with stress-associated transcription factors, including c-MYC, nuclear factor-B (NF-B), and hypoxia-inducible factor 1 (HIF1), resulting in acquired endocrine resistance.

In a paradoxical manner, E2 induces apoptosis in endocrine-resistant breast cancer by activating the secondary UPR via ER.

Specifically, PERK is essential for apoptosis induction, whereas IRE1 and ATF6 are involved in endoplasmic reticulum stress-associated degradation following E2 treatment.

In addition, persistent activation of PERK degrades stress responses in mitochondria and activates the NF-B/tumor necrosis factor (TNF) axis, thereby determining the cell’s apoptotic fate.

The discovery of E2-induced apoptosis has clinical significance for the treatment of breast cancer resistant to endocrine therapy.

Resistant breast and prostate cancers continue to be a major clinical problem; consequently, new therapeutic approaches and more accurate predictors of therapeutic response are required. Due to the involvement of the unfolded protein response (UPR) in cell proliferation and apoptosis evasion, an increasing number of publications, as presented by Direito [119] support the hypothesis that dysfunctions in this network cause and/or aggravate cancer. In addition, UPR activation may contribute to the emergence of drug-resistant phenotypes in breast and prostate cancers. Consequently, targeting this pathway has recently emerged as a promising anticancer treatment strategy.

#### 3.6.7. Proteolytic Neoepitopes for RAS-Driven Cancers

Extracellular proteolysis is frequently dysregulated in disease and can generate proteoforms with neoepitopes that are absent from healthy tissue.

An analysis by [120] demonstrated that antibodies that selectively recognize a proteolytic neoepitope on CUB domain containing protein 1 (CDCP1) may allow for more effective and safer treatment of solid tumors.

CDCP1 is overexpressed and its ectodomain is cleaved by extracellular proteases in RAS-driven cancers, such as breast cancer.

Targeting proteolytic neoepitopes may serve as an orthogonal “AND” gate for enhancing the therapeutic index, an observation even more clinically important as EGFR/RAS pathway activation is prevalent in breast tumors with poor prognosis.

#### 3.6.8. Coumarinyl Thiazolotriazoles

CDK4 and CDK6 are essential regulators of the initial phases of the cell cycle and are a promising anti-cancer treatment option. Structure-based rational design and synthesis of a new class of 1,2,3-triazole-tethered acridinedione derivatives (6a-l) as selective CDK4/6 inhibitors were presented in a study by Praveenkumar [121]. From the entire series of conjugated hexahydro acridinediones, compound 6 g exhibited the most potent cytotoxic effect. Moreover, in a subline of xenograft mouse models, molecule 6 g suppressed tumor growth with fewer adverse effects, suggesting that it could be considered as a novel chemotherapeutic candidate for further comprehensive preclinical breast cancer research.

A study presented by Khan [122] synthesized a series of coumarinyl thiazolotriazoles with varied functional group tolerance and their anticancer properties were evaluated against cancer cell lines (HeLa and MCF-7) and a normal cell line (BHK-21). The results suggested that one of the compounds possesses chemotherapeutic properties against breast cancer and cervical adenocarcinoma cells, necessitating additional research to evaluate this compound’s anticancer efficacy at the clinical level.

#### 3.6.9. IL-25

Immune cell-derived factors, such as cytokines and chemokines, play a pivotal role in the progression of cancer. As a member of the IL-17 cytokine subfamily, IL-25 plays a paradoxical role in cancer, both promoting and inhibiting tumor growth.

Gowhari Shabgah [123] shows that utilizing IL-25-enhancing approaches, such as Virulizin^®^ (mixture of proteins and peptides as immune response modifiers) and dihydrobenzofuran administration, has potentially inhibited tumor cell growth in cancers in which IL-25 has a tumor-suppressive function. In the case of IL-25-dependent tumor progression, however, the use of IL-25 blocking methods, such as anti-IL-25 antibodies, may be complementary to the other anticancer agent. Collectively, it is hoped that IL-25 could be a promising cancer treatment target.

#### 3.6.10. ILK/YAP Axis

Metastasis is the first among the causes of death in cancer patients. In view of and by trying to oppose that phenomenon, the Epithelial-to-Mesenchymal Transition (EMT), a crucial process in cancer metastasis, became a well-established drug development target. LFG-500, a novel synthetic flavonoid with multiple activities, including modulation of EMT in the inflammatory microenvironment, has been indicated as a potential antitumor agent. Using transforming growth factor beta (TGF)-induced EMT models, a study by Li [124] discovered that LFG-500 inhibits migration and invasion associated with EMT in human breast cancer. These results support the use of LFG-500 in cancer treatment and bring consistent proof that the ILK/YAP axis is a reliable biomarker of cancer progression and a novel target for repression of EMT and tumor spread.

#### 3.6.11. Antibody-Drug Conjugates and VEGF Receptor Inhibitors

Despite improvements made to conventional chemotherapies, their use is restricted by a narrow therapeutic window due to off-target toxicities. Antibody-drug conjugates (ADCs) consist of an antibody covalently coupled to a toxic payload via a chemical linker. They provide an elegant solution to the limitations of conventional chemotherapeutics by selectively delivering a highly toxic payload directly to target cells, thereby increasing the efficacy of the delivered cytotoxic while simultaneously reducing systemic exposure and toxicity. Each individual component of an ADC, including the target, the antibody, the linker, and its conjugation chemistry, as well as the cytotoxic payload, must be optimized for maximum efficacy.

In a study presented by Marme [125], it was shown that there are currently nine approved ADCs, including three for breast cancer. With over 100 candidates in various stages of clinical development, the rate of development appears to be quickening.

Cha [126] looks at the expression of the receptor tyrosine kinase ephrin receptor A10 (EphA10), which is undetectable in most normal tissues and at the correlation with tumor progression and poor prognosis in a number of malignancies, including triple-negative breast cancer (TNBC). The conclusion was that targeting EphA10 with EphA10 monoclonal antibodies (mAbs) and EphA10-specific chimeric antigen receptor-T cell therapy may be a promising strategy for patients with EphA10-positive tumors.

Yang, in an analysis of the VEGFR inhibitors finds VEGFR3 to be more 100 times more selective than VEGFR1 and 2, by showing a selective inhibition of proliferation and migration of the cancer cells.

## 4. Discussion

Male breast cancer’s (MBC’s) causes, optimal treatments, and medical/psychosocial consequences are poorly understood. Known risk factors include *BRCA2* mutations (80 times the risk of the general population), and 11 other gene mutations. Other factors implicated are age, conditions that alter the estrogen/androgen ratio, and radiation. Male breast cancer presents typically as a painless retroareolar mass requiring further evaluation. When initial radiological findings are ambiguous or suspicious, more imaging methods are frequently necessary to localize and characterize the lesion and to guide biopsy (core biopsy/fine needle aspiration). 

The global landscape of treatment standards for male patients with breast cancer is not context-appropriate. A call for equality in breast cancer care can be pursued in two divergent ways: a. a gender-neutral delivery of breast cancer information, and b. the recruitment in randomized controlled trials of both sexes. 

## Data Availability

Not applicable.
